# The protective effect of *Ocimum sanctum* leaf extract against lead acetate-induced nephrotoxicity and hepatotoxicity in mice (*Mus musculus*)

**DOI:** 10.14202/vetworld.2021.250-258

**Published:** 2021-01-27

**Authors:** Wiwik Misaco Yuniarti, Nina Krismaharani, Priska Ciptaningsih, Kristania Celia, Kharisma Dwi Veteriananta, Anwar Ma’ruf, Bambang Sektiari Lukiswanto

**Affiliations:** 1 Department of Veterinary Clinic, Faculty of Veterinary Medicine, Universitas Airlangga, Jl. Mulyorejo, Kampus C Unair, Surabaya, 60115, Indonesia; 2 Student of Veterinary Medicine Study Program, Faculty of Veterinary Medicine, Universitas Airlangga, Jl. Mulyorejo, Kampus C Unair, Surabaya, 60115, Indonesia; 3 Department of Basic Veterinary Science, Faculty of Veterinary Medicine, Universitas Airlangga. Jl. Mulyorejo, Kampus C Unair, Surabaya, 60115, Indonesia

**Keywords:** kidney, lead acetate, liver, mice, *Ocimum sanctum*

## Abstract

**Aim:**

The liver and kidneys are the most sensitive organs to lead exposure. Drugs that inhibit the actions of lead in the liver and kidneys are required to protect them from such an exposure. This study investigates the protective effect of the leaf extract of *Ocimum sanctum* (OS) against lead acetate-induced nephrotoxicity and hepatotoxicity in mice.

**Materials and Methods:**

A total of 20 male mice were divided into five equal groups for the 24-day testing period. The negative control group was administered Tween-80 1% orally for 24 days. The positive control group was administered Tween-80 1% orally for 24 days and, starting on day 4, 20 mg/kg BW lead acetate orally once a day for 21 days 1 h after the administration of Tween-80 1%. The other three treatment groups were administered BW OS leaf extract orally in the amount of 140, 280, and 560 mg/kg once a day for 24 days and, starting on day 4, 20 mg/kg BW lead acetate orally for 21 days 1 h after the administration of OS leaf extract. On day 25, the mice were sacrificed to assess the levels of blood urea nitrogen (BUN), creatinine, malondialdehyde (MDA), serum glutamic-oxaloacetic transaminase (SGOT), and serum glutamic-pyruvic transaminase (SGPT) as well as the histopathological changes.

**Results:**

The OS leaf extract caused a decrease in the scores for hepatocyte degeneration and portal inflammation (p<0.05) but not for hepatic necrosis (p>0.05) in mice exposed to lead. Similar patterns were observed in the effect of OS leaf extract on the renal morphofunction. The OS leaf extract decreased the scores for hydropic degeneration, tubular necrosis, and glomerular necrosis. The levels of MDA, SGOT, SGPT, BUN, and creatinine decreased in the lead-exposed mice treated with OS leaf extract (p<0.05).

**Conclusion:**

The administration of OS leaf extract has a protective effect against lead acetate-induced hepatotoxicity and nephrotoxicity in mice.

## Introduction

Lead is a toxic metal that can occur in air, water, and soil. It has caused extensive environmental contamination and health problems in numerous parts of the world [1]. Lead exposure is a global health concern as this toxic metal is found in the environment, including agricultural areas, as well as in contaminated food and soil as a result of industrial pollution [2]. It is considered to be the most dangerous natural heavy metal as it causes metabolic dysfunction in human and animal organ systems [3]. The mechanism of lead toxicity is oxidative stress, through which lead increases the productivity of free radicals and reactive oxygen species (ROS) and directly suppresses the body’s antioxidant system [4].

Lead interferes with the thiol content of erythrocytes and exerts an effect on sulfhydryl-containing enzymes, antioxidant defenses, and tissues rich in mitochondria, thus causing clinical syndrome symptoms [5]. Oxidative stress is caused by ROS imbalance and the ability of a biological system to readily detoxify the reactive intermediates that can cause lipid peroxidation, which leads to the formation of various active compounds and results in cellular damage [6].

Autopsy studies of lead-exposed animals indicate that the liver is the largest lead repository (33%) among soft tissues, followed by the kidneys [7]. When lead is absorbed through the portal system, the first organ that is exposed is the liver. Lead causes liver damage by inducing the formation of free radicals that reduce the efficiency of the body’s antioxidant system; thus, oxidative stress naturally occurs [8]. The accumulation of significant amounts of lead in the liver tissue is implicated in the induction of oxidative stress responses in the liver [7,9]. One of the effects of ROS that can interfere with renal physiology and cause lead toxicity is nephrotoxicity. This is due to the absorption ability of the kidneys during filtration and reabsorption; thus, the kidneys become a site where divalent metal ions accumulate [10]. According to the same researchers, lead reacts with cell junctions to change the structure of the epithelial cells; thus, the lumen of the proximal tubules in the kidneys shrink as a result of oxidative stress [11].

The occurrence of liver and kidney damage due to lead exposure can be detected by assessing the levels of malondialdehyde (MDA), serum glutamic-oxaloacetic transaminase (SGOT), serum glutamic-pyruvic transaminase (SGPT), blood urea nitrogen (BUN), and serum creatinine.

Previous study suggests that the administration of various natural antioxidants could prevent and cure the toxic effects of lead that cause the generation of free radicals [12]. In Indonesia, the genus *Ocimum* (commonly known as “basil” or “tulsi”) is very useful due to its curative potential. *Ocimum sanctum* (OS) leaves have several properties, including anti-inflammatory, anti-allergic, radioprotective, anticarcinogenic, and antioxidant [13]. OS contains a wide range of essential oils rich in phenolic compounds and other natural products of great pharmacological importance for antioxidant use, such as flavonoids and anthocyanins [14]. Moreover, it contains phenols, flavonoids, carotenoids, ascorbic acid, riboflavin, and thiamine [15,16], as well as Vitamins E and A [17].

Due to the hepatotoxic and nephrotoxic effects caused by exposure to lead acetate, and due to the antioxidant activity of the OS leaf extract, this study examines the protective effect of the OS leaf extract against lead acetate-induced hepatotoxicity and nephrotoxicity in mice (*Mus musculus*).

## Materials and Methods

### Ethical approval

The Animal Care and Use Committee Fakultas Kedokteran Hewan Universitas Airlangga Surabaya has approved experimental protocol that the research could be done with the treatment that does not violate medical ethic of animal welfare. Ethical clearance declaration was published with certificate number: 1.KE.179.10.2019.

### Study period and location

This research was conducted at the Faculty of Veterinary Medicine, Universitas Airlangga, Surabaya, from January to March 2020.

### Animals

The experimental animals used in this study were healthy male mice aged 2.5-3 months, with an average body weight of 25-30 g. They were obtained from Pusat Veterinaria Farma Surabaya and were then fed and underwent environmental adaptation for 7 days. The experiment conducted has a completely randomized design. A total of 20 male mice were divided into five equal groups for the 24-day testing period. The animals were housed in plastic cages, fed standard commercial mice chow, and provided drinking water *ad libitum*.

### Chemicals

The research materials used were lead acetate (Sigma-Aldrich, Indonesia), (Tween-80 1%) (Sigma-Aldrich, Indonesia), mice feed, drinking water, ketamine (Ilium, Australia), xylazine (Ilium, Australia), and OS leaf extract that was processed using ethanol 96% pro analysis (Smartlab, Indonesia). Thiobarbituric acid (TBA) was the reagent used to evaluate the MDA levels of the liver and kidney. Assay was successfully performed using physiological saline (NaCl 0.9%), aquadest, trichloroacetic acid 10% (Sigma-Aldrich, Indonesia), 1N HCL (Sigma-Aldrich, Indonesia), Na-thiobarbiturate 1% (Sigma-Aldrich, Indonesia), and standard solution. The reagents used to measure the SGOT level were reagent 1 (Tris buffer solution [pH 7.8], L-aspartate, LDH, and MDH) and reagent 2 (CAPSO, 2-oxoglutarate, and NADH) (Erba Mannheim, Germany). The reagents used to measure the SGPT level were reagent 1 (Tris buffer solution [pH 7.5], L-alanine, and LDH) and reagent 2 (CAPSO, 2-oxoglutarate, and NADH) (Erba Mannheim, Germany). The slides used to measure the histopathological changes in the liver and kidney were prepared using formaldehyde buffer 10%, alcohol (70%, 80%, 90%, and 96%), xylol, paraffin, and hematoxylin and eosin (H&E) stain. The reagents required to measure the BUN level were reagent 1 (Tris buffer solution 100 mmol/L, α-ketoglutarate 5.49 mmol/L, urease 10 kU/L, and urease glutamic dehydrogenase [GLDH] 3.8 kU/L) and reagent 2 (NADH 1.66 mmol/L) Diasys Diagnostic System GmbH, Germany. The reagents used to measure the serum creatinine level were reagent 1 (sodium hydroxide 0.24 mol/L) and reagent 2 (picric acid 26 mmol/L) Diasys Diagnostic System GmbH, Germany.

### Preparation of the OS leaf extract

The OS leaves were collected from Sleman, Daerah Istimewa Yogyakarta, Indonesia. They were washed with tap water, chopped, and dried at room temperature and normal humidity. The leaves were then weighed and blended to a fine powder to obtain the leaf extract. The extraction process was performed at the Pharmacognosy Laboratory, Faculty of Pharmacy, Universitas Airlangga, Surabaya. The powder (500 g) was macerated with ethanol 96% for 3 days. Then, the filtrate was collected and allowed to evaporate using a rotary evaporator at 40°C until a half-condensed ethanol extract of the OS leaves was produced [18]. The extract was stored at 0-4°C and used for the experiment in a Tween-80 1% suspension.

### Experimental design

Twenty male mice were divided into five groups. They were treated orally with OS leaf extract [18] as follows:

C(−): Mice were administered Tween-80 1% (24 days) and aquadest (starting on day 4 for 21 days)

C(+): Mice were administered Tween-80 1% (24 days) and lead acetate 20 mg/kg BW (starting on day 4 for 21 days)

T1: Mice were administered OS leaf extract 140 mg/kg BW (24 days) and lead acetate 20 mg/kg BW (starting on day 4 for 21 days)

T2: Mice were administered OS leaf extract 280 mg/kg BW (24 days) and lead acetate 20 mg/kg BW (starting on day 4 for 21 days)

T3: Mice were administered OS leaf extract 560 mg/kg BW (24 days) and lead acetate 20 mg/kg BW (starting on day 4 for 21 days)

### Sampling

On day 25, the mice were euthanized by intraperitoneal injection of a combination of ketamine (100 mg/kg BW) and xylazine (10 mg/kg BW) [19]. The thorax and abdomen were dissected, and the left lobe of the liver and the left kidney were taken to measure the MDA level. Liver and kidney were immediately washed, placed in NaCl 0.9% solution, and stored at −25°C until MDA assay. The right lobe of the liver and the right kidney were collected for the analysis of the histopathological changes and fixed in 10% neutral buffer formalin. Blood sample (1.5 mL) was obtained through cardiac puncture to evaluate the levels of SGOT, SGPT, BUN, and serum creatinine.

The MDA level of the liver and kidney was determined spectrophotometrically through TBA assay. The reaction of TBA and MDA in the solution led to the formation of a red chromogen TBA–MDA complex that can be colorimetrically measured using a spectrophotometer with a 532-nm wavelength. Sodium chloride 0.9% (200 μL), aquadest (550 μL), and trichloroacetic acid 10% (100 μL) were added to 100 mg of ground liver and homogenized through vortex. Subsequently, 1N HCl (250 μL) and Na-thiobarbiturate 1% (100 μL) were added and also homogenized through vortex. The mixture was incubated in a 100°C water bath for 20 min and then allowed to cool. It was then centrifuged at 500 rpm for 10 min. The supernatant (300 μL) was obtained and measured using a spectrophotometer with a 532-nm wavelength. The value of absorbance was measured by comparing against a blank that contains all the reagents minus samples homogenate [20].

The blood stored in a plain vacuum tube was centrifuged at 3000 rpm for 10 min to obtain the serum samples required for the measurement of the SGOT, SGPT, BUN, and serum creatinine levels. The serum samples were placed in Eppendorf tubes. The SGOT, SGPT, BUN, and serum creatinine levels were measured through photometry using a clinical chemical analyzer (ERBA Mannheim GmbH XL 600) with a 340-nm wavelength.

The SGOT level was measured after the addition of two reagents. Reagent 1 was mixed with the sample and incubated at 37°C for 1 min. Next, reagent 2 was added, and the sample was incubated again at 37°C for 1 min. The initial absorbance of the calibrator and the sample was measured against a blank reagent. The change in the absorbance was measured at 1, 2, and 3 min (ΔA/min).

The BUN level was measured through the urease GLDH method using two reagents. The serum creatinine level was measured using the Jaffe reaction method after the addition of two reagents. Reagent 1 was added, and the mixture was incubated at 37°C for 1 min; subsequently, reagent 2 was added. The absorbance results were calculated using the difference between the initial and final absorbance.

### Histopathological study

The livers and kidneys fixed in 10% neutral buffered formalin were paraffin embedded, and the obtained serial sections were stained with H&E. The stained sections were examined under a light microscope. Microscopic observation was completed at magnifications of 100×, 200×, and 400× to analyze the histopathological changes. The observed grades of the pathological changes in the kidneys were based on the modified Klopfleisch [21] scoring method. The histopathological changes in the kidney were tubular epithelial hydropic degeneration, tubular epithelial necrosis, and glomerular necrosis. The grades of the pathological changes in the liver were based on the scoring method by Knodell *et al*. [22], which consisted of hydropic degeneration, necrosis, and portal inflammation.

### Statistical analysis

Data were expressed as means ± standard deviations, and the results were analyzed using the IBM^®^ SPSS software version 23 (IBM Corp, Armonk, New York, USA). One-way ANOVA was conducted, and statistical comparisons among the groups were performed using Tukey’s Honestly Significant Difference test for the MDA levels of the liver and kidney and Duncan’s Multiple Range Test for the SGOT, SGPT, BUN, and serum creatinine levels. The end data of the histopathological changes in the kidney and liver were analyzed using the Kruskal–Wallis test to determine the most effective dose and the Mann–Whitney U-test for the statistical comparison.

## Results

### Effects of the OS leaf extract on lead acetate-induced hepatotoxicity

#### Effects on the histopathological changes in the liver

**Figure-1:**
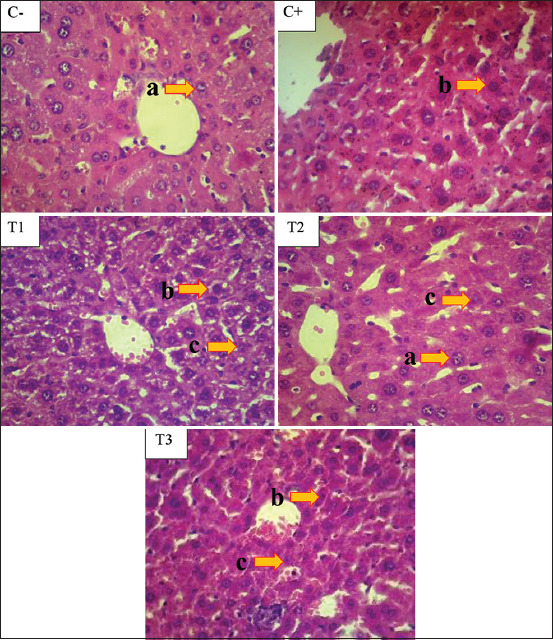
Histological study of pretreatment with *Ocimum sanctum* leaf extract on lead acetate induced hepatotoxicity in mice liver. Normal morphology of mice liver in C(−) showed by normal appearance of hepatocyte (a). The necrotic hepatocyte (b) showed in lead acetate treated group of C(+). Treated group with 140 mg/kg BW (T1), 280 mg/kg BW (T2), and 560 mg/kg BW (T3) of *Ocimum sanctum* showed the appearance of degenerative hepatocyte (c). H&E 400×.

**Figure-2:**
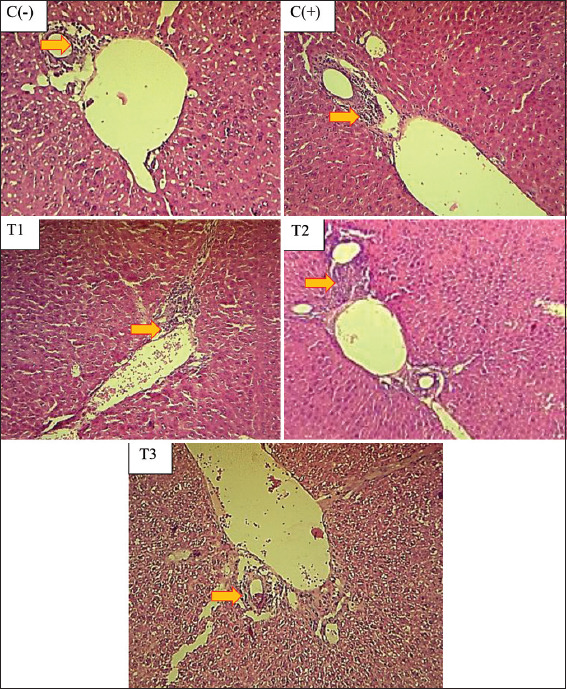
Portal inflammation in portal vein of mice liver indicated by the appearance of inflammation cell (indicated by arrows). C(−): Control negative, C(+): Control positive (20 mg/kg BW lead acetate), T1: 140 mg/kg BW *Ocimum sanctum* + lead acetate, T2: 280 mg/kg BW + lead acetate, T3: 560 mg/kg BW *Ocimum sanctum* + lead acetate. H&E 200×.

The H&E-stained slide sections of mice liver from the control negative group exhibited normal characteristics of the hepatic structure. The hepatic lobules appeared to be made up of hepatocytes arranged in cords radiating from a central vein. The livers of the lead acetate-treated group (positive control) demonstrated tissue structural changes by the presence of hepatic necrosis. A significant difference (p<0.05) was observed between the positive control group and the negative control group. The positive control group did not exhibit a significant increase (p>0.05) in hydropic degeneration or portal inflammation when compared with the negative control group or the pretreated groups (T1, T2, and T3). The necrosis observed in the positive control group and the three pretreated groups was significantly different from that observed in the negative control group (p<0.05) (Table-1 and Figures 1and 2).

**Table-1:** Mean±SD of mice (*Mus musculus)* liver histopathological changes from each treatment.

Group	Mean±SD		

	Hydropic degeneration	Necrosis	Portal inflammation
C(−)	1.00^a^±0.00	1.33^a^±2.30	1.67^a^±1.52
C(+)	2.67^a^±1.52	7.33^b^±1.15	2.67^a^±0.57
T1	1.33^a^±0.57	4.67^b^±1.15	1.67^a^±1.15
T2	1.00^a^±0.00	6.00^b^±0.00	1.33^a^±1.52
T3	2.33^a^±1.15	6.67^b^±3.05	2.00^a^±1.00

Description: ^a,b^Different superscript in the same column shows significant differences (p<0.05)

#### Effects on the liver MDA, SGOT, and SGPT levels

The positive control group demonstrated a significant (p<0.05) increase in the MDA levels when compared with the negative control group. The T3 group, treated with 560 mg/kg BW OS leaf extract, exhibited a significant (p<0.05) decrease in the liver MDA levels when compared with the positive control group.

The positive control group exhibited the highest MDA, SGOT, and SGPT levels, whereas the negative control group exhibited the lowest levels. The SGOT evaluation between the negative control group and the three pretreated groups (T1, T2, and T3) did not significantly differ (p>0.05). Moreover, no significant difference was observed between the positive group and Groups T1 and T3. Significant differences (p<0.05) in the SGOT levels were observed between the negative control and T2 groups when compared with the positive control group. The SGPT analysis revealed no significant difference (p>0.05) between the negative control group and T2 group or between the positive control group and the three pretreated groups. Conversely, a significant difference (p<0.05) was observed when the negative control group was compared with the positive control, T1, and T3 groups (Table-2).

**Table-2:** Mean±SD of mice (*Mus musculus*) liver MDA, SGOT, and SGPT level from each treatment.

Group	Mean±SD		

	Liver MDA (nmol/g)	SGOT (U/L)	SGPT (U/L)
C(−)	218.00^a^±50.16	144.50^a^±25.98	90.25^a^±13.94
C(+)	438.50^c^±27.57	229.00^b^±56.79	121.00^b^±12.11
T1	427.25^c^±25.91	196.50^ab^±36.30	119.25^b^±28.54
T2	369.25^bc^±32.25	166.25^a^±24.12	94.75^ab^±7.09
T3	310.00^b^±33.69	184.50^ab^±7.72	119.00^b^±10.80

Description: ^a,b,c^different superscript in the same column shows significant differences (p<0.05). BUN=Blood urea nitrogen, MDA=Malondialdehyde, SGPT=Serum glutamic-pyruvic transaminase, SGOT=Serum glutamic-oxaloacetic transaminase

### Effects of the OS leaf extract on lead acetate-induced nephrotoxicity

#### Effect on the kidney histopathological changes

**Figure-3:**
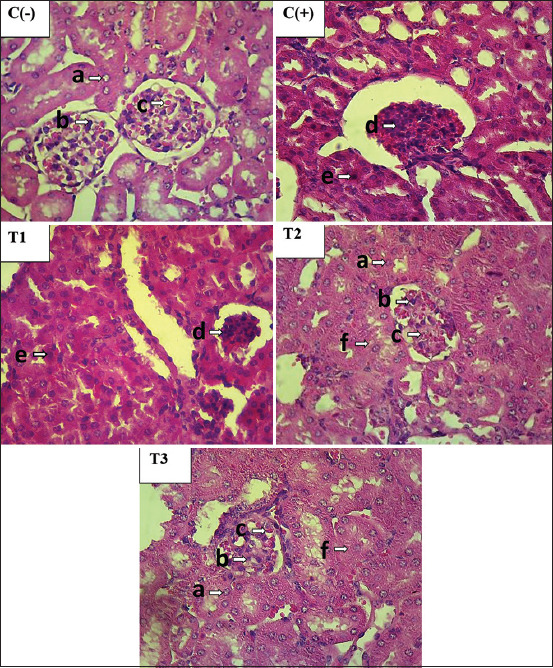
Histological study of pretreatment with *Ocimum sanctum* leaf extract on lead acetate induces nephrotoxicity in mice kidney. Normal morphology of mice kidney in C(−) showed by normal tubular epithelial cell (a) and glomerulus indicated by the appearance of normal mesangial cell (b) and capillary lumen (c). The lead acetate treated group C(+) and 140 mg/kg BW of *Ocimum sanctum* (T1) showed necrosis in mesangial cell (d) and tubular epithelial cell (e). Mice treated with 280 mg/kg BW (T2) and 560 mg/kg BW (T3) of *Ocimum sanctum* showed the appearance of hydropic degeneration (f) in tubular epithelial cell. H&E 400×.

Microscopic examination of healthy kidneys revealed normal tubular epithelial cells and glomerulus without any structural changes in the tissues. Exposure to lead acetate generated massive hydropic degeneration and necrosis (pyknosis) in the tubular epithelial cells, especially in the proximal convoluted tubule cells. Meanwhile, the glomerulus exhibited severe necrosis in the mesangial cells. Pretreatment with the OS leaf extract significantly prevented the histopathological changes. The positive control group demonstrated structural changes in the kidney tissue, such as hydropic degeneration, necrotic tubular cells, and glomerular necrosis. The exposure of the positive control group to lead acetate caused a significant difference (p<0.05) in the histopathological changes when compared with the negative control group. Administration of the OS leaf extract to T1, T2, and T3 groups reduced the degree of hydropic degeneration, tubular necrosis, and glomerular necrosis. Moreover, the three treated groups had significantly reduced levels of MDA, BUN, and creatinine when compared with the positive control group (p<0.05). The OS leaf extract in the pretreated groups mitigated cell destruction, which caused significant differences (p<0.05) in the histopathological changes (particularly hydropic degeneration and necrotic tubular cells) when compared with the positive control group, trending toward normal (the results of the negative control group) (Table-3 and Figure-3).

**Table-3:** Mean±SD of mice (*Mus musculus)* kidney histopathological changes from each treatment.

Group	Mean±SD		

	Hydropic degeneration	Necrotic tubular cells	Glomerular necrosis
C(−)	0.50^a^±0.57	1.00^a^±1.15	0.75^a^±1.50
C(+)	2.75^b^±0.95	4.50^b^±1.91	5.00^b^±2.82
T1	2.75^b^±0.50	3.50^ab^±1.91	4.00^b^±1.55
T2	2.50^b^±0.57	2.50^ab^±1.00	2.75^ab^±2.06
T3	1.25^a^±0.50	1.50^a^±1.00	2.00^ab^±2.44

Description: ^a,b^different superscript in the same column shows significant differences (p<0.05)

#### Effect on the kidney MDA, BUN, and serum creatinine levels

Administration of 20 mg/kg BW lead acetate for 21 days to the positive control group did not result in significant increases (p>0.05) in the levels of serum creatinine and BUN when compared with the negative control group. Level of MDA in the positive control group was significantly higher compared with the negative control group (p<0.05). Reductions in the MDA level were observed in the OS leaf extract-pretreated groups. These groups demonstrated a significant decrease (p<0.05) in the BUN and serum creatinine levels when compared with the positive control group. However, no significant difference (p>0.05) was observed in the MDA, BUN, and serum creatinine levels among the T1, T2, and T3 groups that received the three different graded dosages (140, 280, and 560 mg/kg BW) (Table-4).

**Table-4:** Mean±SD of mice (*Mus musculus*) kidney MDA, BUN, serum creatinine level from each treatment.

Group	Mean±SD		

	Kidney MDA (nmol/g)	BUN (mg/dl)	Creatinine (mg/dl)
C(−)	269.67^a^±80.41	26.28^b^±1.27	0.40^ab^±0.04
C(+)	494.00^b^±66.57	27.63^b^±3.34	0.45^b^±0.07
T1	423.33^ab^±56.58	19.30^a^±3.85	0.36^a^±0.04
T2	361.33^ab^±42.66	20.05^a^±3.52	0.33^a^±0.04
T3	343.00^ab^±78.93	18.13^a^±1.35	0.36^a^±0.06

Description: ^a,b^different superscript in the same column shows significant differences (p<0.05). MDA=Malondialdehyde, BUN=Blood urea nitrogen

## Discussion

Lead acetate enhanced the intracellular formation of ROS causing liver and kidney damage. Under the influence of lead, oxidative stress occurs through two different pathways: (1) The generation of ROS, such as hydrogen peroxide, singlet oxygen, and hydrogen peroxide, and (2) the depletion of antioxidant reserves [23]. Oxidative stress occurs when there is an increased production of free radicals or ROS, such as singlet oxygen, superoxide, hydroxyl radicals, hydrogen peroxide, and hydroperoxyl radicals while the antioxidant enzymes in the body are decreased [24]. ROS attack all the cellular structures, which results in the loss of cellular and mitochondrial membrane integrity as the membrane polyunsaturated fatty acids (PUFA) undergo an intensified process of lipid peroxidation [25,26]. This study revealed various degrees of histological changes accompanied by biochemical changes in the liver and kidney tissues.

During oxidative stress, the overproduction of free radicals had a negative effect on cells, tissues, inflammatory responses, and apoptosis [27]. Thus, when the rate of free radicals in the body increases, oxidative stress occurs, resulting in the dysregulation of hepatic homeostasis, which leads to hepatic injury [28]. Microscopic examination of the histological changes in the liver revealed a disturbed hepatic architecture. The hepatic necrosis that occurred due to exposure to 20 mg/kg BW lead acetate may indicate oxidative stress on hepatocytes. ROS induce stressful situations at the cellular level and cause structural damage to cells, proteins, nucleic acids, membranes, and lipids [11]. This may lead to necrosis, hydropic degeneration, and portal inflammation of the liver cells.

MDA is one of the products of membrane PUFA peroxidation and is responsible for cell membrane damage [29]. It is also one of the biomarkers used to determine oxidative stress levels in clinical situations. Increased MDA levels indicate increased lipid peroxidation in the liver, which leads to tissue damage and the failure of antioxidant mechanisms in preventing the formation of excessive free radicals [30].

SGOT and SGPT are aminotransferase group enzymes that act as indicators of damage in the liver cells. In this study, an increase in the SGPT levels followed by the SGOT levels may indicate that administration of 20 mg/kg BW lead acetate for 21 days can disrupt the morphological structure of hepatocytes. PUFAs, which are contained in the phospholipid membrane, are the materials most often involved in the oxidation mechanism as they contain numerous double bonds between molecules [31]. Lipid peroxidation increases the permeability of the cell membrane and disrupts the distribution of ions, causing damage to the cell membrane and organelles. SGPT, a hepatocyte cytoplasmic enzyme, is released to the circulatory system when cell membrane damage occurs. The magnitude of the increase in SGPT activity indicates the level of hepatocyte damage [32]. The mitochondria in the liver cells are also susceptible to damage from oxidative stress, and when this damage occurs, the SGOT levels increase. In the liver cells, SGOT is located in the mitochondria and cytosol. An increase in the SGOT levels could indicate the occurrence of non-hepatic abnormalities and liver damage, dominated by mitochondrial damage. If the cells are damaged, these enzymes leak into the circulatory system, and their blood serum levels increase.

The kidneys are organs susceptible to ROS-induced cellular injuries due to the abundance of PUFAs in their cellular compositions [33]. Lipid peroxides are a form of peroxyl radicals produced as a result of PUFA peroxidation. They have the ability to generate MDA as a decomposition product. MDA is potentially harmful to other cellular macromolecules, such as protein and nucleic acids. The interaction between MDA and nucleic acids may result in the alteration in the DNA structure and gene expression as well as the formation of cancerous cells [33].

The renal cortex is more affected than the medulla as 90% of the total renal blood flow enters the cortex through the blood stream. Thus, high concentrations of lead acetate mainly affect the cortex rather than the medulla [34]. The proximal convoluted tubules suffer the most damage from lead acetate exposure as they reabsorb 60-80% of the glomerular filtration product [35]. In this study, tubular epithelial hydropic degeneration and tubular epithelial necrosis demonstrated significant differences, whereas glomerular necrosis did not. This is because lead acetate mainly targets the proximal convoluted tubules rather than the glomerulus. Hydropic degeneration is a common expression of cell injury as there is an influx of water and sodium ions (when sodium–potassium ion pumps fail) that cause swelling [36]. The research conducted by Sharma and Singh indicated that when Balb-c mice were exposed to lead acetate, tubular changes occurred earlier than glomerular and interstitial changes, and the incidence and severity of the histopathological changes increased along with the amounts of dose. Moreover, tubular damage was more prominent in the proximal than in the distal convoluted tubules [37].

Increases in the serum creatinine and BUN levels are considered signs of impaired kidney function as the kidneys are not able to maintain normal ranges of these waste products. This finding is consistent with that of the study conducted by Rana *et al*. [10], who found that lead can react with cell junctions, thus changing the structure of the epithelial cells. This causes the lumen of the proximal tubules in the kidneys to shrink as a result of oxidative stress. Impaired membrane permeability may cause leakage, allowing urea, and creatinine enter back into the blood stream. Increased levels of serum creatinine and BUN, due to exposure to lead acetate, may occur due to necrosis and progressive alterations of the glomerulus and tubules [38,39].

OS, which is commonly known as holy basil, is an aromatic herb belonging to the family Lamiaceae. OS leaves contain 0.7% volatile oil and comprised of approximately 71% eugenol [40] as well as ascorbic acid, Vitamin A, phenolics, flavonoids, riboflavin, and minerals [41]. Eugenol was found to be the predominant phenolic compound in the OS extract and acts as a potential source of antioxidants exhibiting free-radical scavenging activity [42,43]. In this study, it was found that administration of OS leaf extract protected the liver and kidney from lead acetate exposure. These findings are consistent with those by Manikandan *et al*. [18] who demonstrated that OS leaf extract could prevent cell damage by inhibiting oxidative stress and lipid peroxidation.

OS leaf extract contains eugenol and flavonoids (orientin and vicenin) that exhibit free radical scavenging activity in the cells [40]. Eugenol exhibits the most powerful antioxidant and radical scavenging activities [44]. Moreover, it prevented radiation-induced chemical oxidative damage in the membranes and modified the membrane-associated signaling process after radiation exposure. The mechanism of eugenol is to transfer a cation hydrogen atom from its hydroxyl group to peroxyl radicals to prevent attacks on cell membrane PUFAs. The data presented by Binu *et al*. [45] confirmed that eugenol could decrease lipid peroxidation and SGOT and SGPT levels in rats demonstrating arsenic-induced toxicity. The flavonoids (orientin and vicenin) obtained from the OS leaf extract significantly reduced radiation-induced lipid peroxidation [46] and prevented oxidative stress in cells by chelating redox-active metal ions and terminating free-radical chain reactions [47]. Ascorbic acid is effective in regenerating the antioxidant form of Vitamin E by reducing tocopherol radicals, thus protecting the membranes and other cell compartments from free-radical damage [48]. Ascorbic acid acts as an antioxidant by transforming into ascorbate radical, by donating an electron to the lipid radical in order to terminate the lipid peroxidation chain reaction. Ascorbic acid also directly scavenges O^2^_-_ and OH_-_ and regenerates oxidized carotenoids. Vitamin A (a carotenoid) deactivates peroxyl radicals by reacting with them to form resonance-stabilized radical adducts and disrupts the reaction sequence to prevent lipid peroxidation [49].

## Conclusion

Treatment of mice with graded dosages of OS leaf extract prevented the levels of MDA, SGOT, glutamic-pyruvic transaminase, BUN, and serum creatinine from increasing when mice were exposed to lead acetate. The antioxidant protective mechanisms of OS leaf extract attenuated oxidative stress and scavenged the free radicals responsible for liver and kidney damage, inhibiting lipid peroxidation in the cell membrane. This prevented cell injury, protein oxidation, and DNA damage in the nucleus. Based on this study, administration of OS leaf extract has a protective effect against lead acetate-induced hepatotoxicity and nephrotoxicity in mice.

## Authors’ Contributions

PC carried out the measurement of liver MDA level. KDV carried out the measurement of SGOT and SGPT level. NK carried out the measurement of BUN and serum creatinine. KC observed the histopathological changes in kidney. WMY, BSL, and AM carried out the measurement of kidney MDA level and observed the histopathological changes in liver. All authors reviewed, commented, and approved the final manuscript.
